# Inflammatory and Antimicrobial Responses to Methicillin-Resistant *Staphylococcus aureus* in an *In Vitro* Wound Infection Model

**DOI:** 10.1371/journal.pone.0082800

**Published:** 2013-12-10

**Authors:** Elisabeth M. Haisma, Marion H. Rietveld, Anna de Breij, Jaap T. van Dissel, Abdoelwaheb El Ghalbzouri, Peter H. Nibbering

**Affiliations:** 1 Department of Infectious Diseases, Leiden University Medical Center, Leiden, The Netherlands; 2 Department of Dermatology, Leiden University Medical Center, Leiden, The Netherlands; University of Central Florida College of Medicine, United States of America

## Abstract

Treatment of patients with burn wound infections may become complicated by the presence of antibiotic resistant bacteria and biofilms. Herein, we demonstrate an *in vitro* thermal wound infection model using human skin equivalents (HSE) and biofilm-forming methicillin-resistant *Staphylococcus aureus* (MRSA) for the testing of agents to combat such infections. Application of a liquid nitrogen-cooled metal device on HSE produced reproducible wounds characterized by keratinocyte death, detachment of the epidermal layer from the dermis, and re-epithelialization. Thermal wounding was accompanied by up-regulation of markers for keratinocyte activation, inflammation, and antimicrobial responses. Exposure of thermal wounded HSEs to MRSA resulted in significant numbers of adherent MRSA/HSE after 1 hour, and multiplication of these bacteria over 24-48 hours. Exposure to MRSA enhanced expression of inflammatory mediators such as TLR2 (but not TLR3), IL-6 and IL-8, and antimicrobial proteins human β-defensin-2, -3 and RNAse7 by thermal wounded as compared to control HSEs. Moreover, locally applied mupirocin effectively reduced MRSA counts on (thermal wounded) HSEs by more than 99.9% within 24 hours. Together, these data indicate that this thermal wound infection model is a promising tool to study the initial phase of wound colonization and infection, and to assess local effects of candidate antimicrobial agents.

## Introduction

The intact skin is protected against microbial invasion by its chemical and physical barrier properties together with the skin microbiome [1]. However, after (thermal) wounding the balance between microbial invasion and these protective mechanisms is disturbed and wounds may easily become colonized by opportunistic bacteria [2] such as Staphylococci [3], *Pseudomonas* [4] and *Acinetobacter* strains [5]. Many of these bacteria can become resistant to current antibiotics and/or form biofilms in which they are protected from the actions of the host immune system and antibiotics [6,7]. Moreover, bacteria colonizing the wound bed trigger an inflammatory response and this may lead to improper wound healing and – if not controlled properly – ultimately to invasive infection and sepsis [8]. Clearly, there is a need to better understand the local conditions favoring colonization and invasive infection, and to develop an *in vitro* model that mimics the initial phases of these processes in humans, and to enable testing of new agents to combat wound infections. 

Murine and porcine (burn) wound models are widely used to study wound infections [9,10]. However, these animal models suffer from serious drawbacks, such as poor representation of the human skin, being laborious and costly, and raise ethical issues. By contrast, human skin equivalents (HSEs) recapitulate most of the characteristics of the intact human skin including a fibroblast populated dermis, a multilayered epidermis, and a competent skin barrier [11,12]. In response to wounding HSE mimics the epithelialization process as found in the *in vivo* situation [13]. Furthermore, HSEs, but not keratinocyte cell monolayers, offer the same specific conditions as the human skin for bacteria to attach to the surface [14]. In this respect, others already have demonstrated that bacteria like *Staphylococcus aureus* (*S. aureus*) and *Staphylococcus epidermidis* can colonize intact HSEs and trigger the expression of pro-inflammatory cytokines/chemokines such as IL-6 and IL-8 by the underlying skin cells [15,16]. 

The aim of this study was to develop an in vitro thermal wound infection model, using the current thermal wound model [13], and study subsequent with methicillin resistant *S. aureus* (MRSA). Next, we evaluated the immunological and antibacterial responses in this thermal wound infection model. Finally, we assessed the potential of this model for screening of new candidate antimicrobial agents by studying the effect of the antibiotic mupirocin on the number of bacteria on these HSE. 

## Results

### Characterization of HSE after standardized thermal wounding

**Figure 1:**
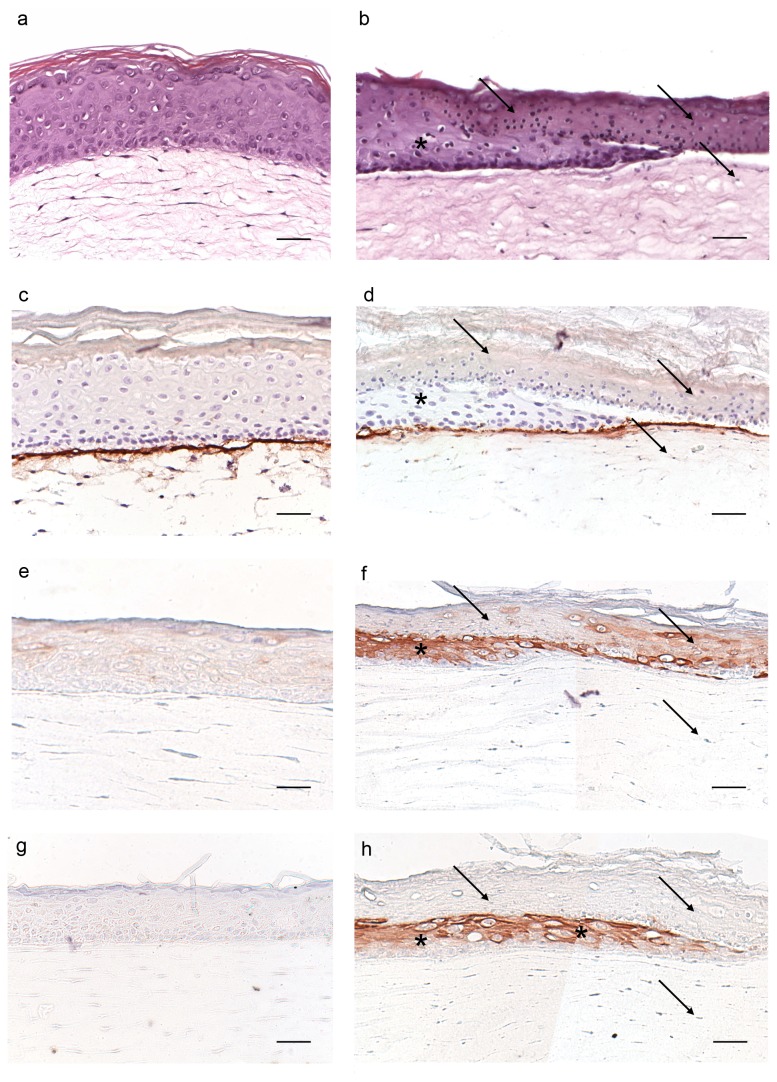
Morphology, basement membrane protein collagen type IV and keratin expression in control and thermal wounded HSEs. Shown are cross-sections of the paraffin embedded control and thermally wounded HSEs that were stained with either (**a**-**b**) hematoxylin and eosin (H&E), or immunolabeled for (**c**-**d**) collagen type IV, (e-f) K16, or (**g**-**h**) K17. Control HSEs (**a**,**c**,**e**,**g**) and HSEs 48 hours after thermal wounding (**b**,**d**,**f**,**h**). Arrows indicate dead epidermis and fibroblasts in the dermis. * indicates re-epithelialization. Pictures are representative for 3 NHK donors. Scale bars = 50 μm.

HSEs were thermally wounded by a device cooled with liquid nitrogen for 15 seconds, as described in detail by El Ghalbzouri et al., 2004. Hereafter cell viability and re-epithelialization were evaluated by microscopy at different time points. Examination of HSEs after 48 hours revealed that wounding results in a dead epidermal layer detaching from the dermal compartment (Figures 1a-b). Beneath the wounded area, fibroblast cell death occurred as judged from the presence of small round-shaped cells (Figure 1b, arrows). Wound healing was observed within the first 48 hours as evidenced by the re-epithelialization (Figure 1b). In intact HSEs, collagen type IV is strongly expressed at the dermal-epidermal junction, while in thermal wounded HSEs this expression is weaker in the re-epithelialization tongue of the HSEs (Figure 1c-d). 

After thermal wounding, messenger RNA (mRNA) levels of keratinocyte activation markers keratin (K) 16 and K17 [17] show a 4.4±0.7 (p=0.071) and 57.9±5.0 (p<0.001) fold increase at 48 hours, respectively, as compared to intact HSEs (n=3). This was confirmed by immunohistochemical staining showing K16 and especially K17 expression being confined to the re-epithelialization tongue (Figure 1e-h). Using Ki67 staining we showed that thermal wounded HSEs had a lower number of proliferating keratinocytes as compared to control HSEs. The number of Ki67 positive cells in the basal layer per microscopical field was 13.8±0.2 cells in thermal wounded versus 10.7±0.4 cells in control HSEs, (p=0.057, n=3), data not shown. Together, thermal wounding of HSEs results in reproducible wounds characterized by a dead epidermis becoming detached from the dermis, and by a wound repair process. 

### Adherence and multiplication of MRSA on thermal wounded and control HSEs

**Figure 2:**
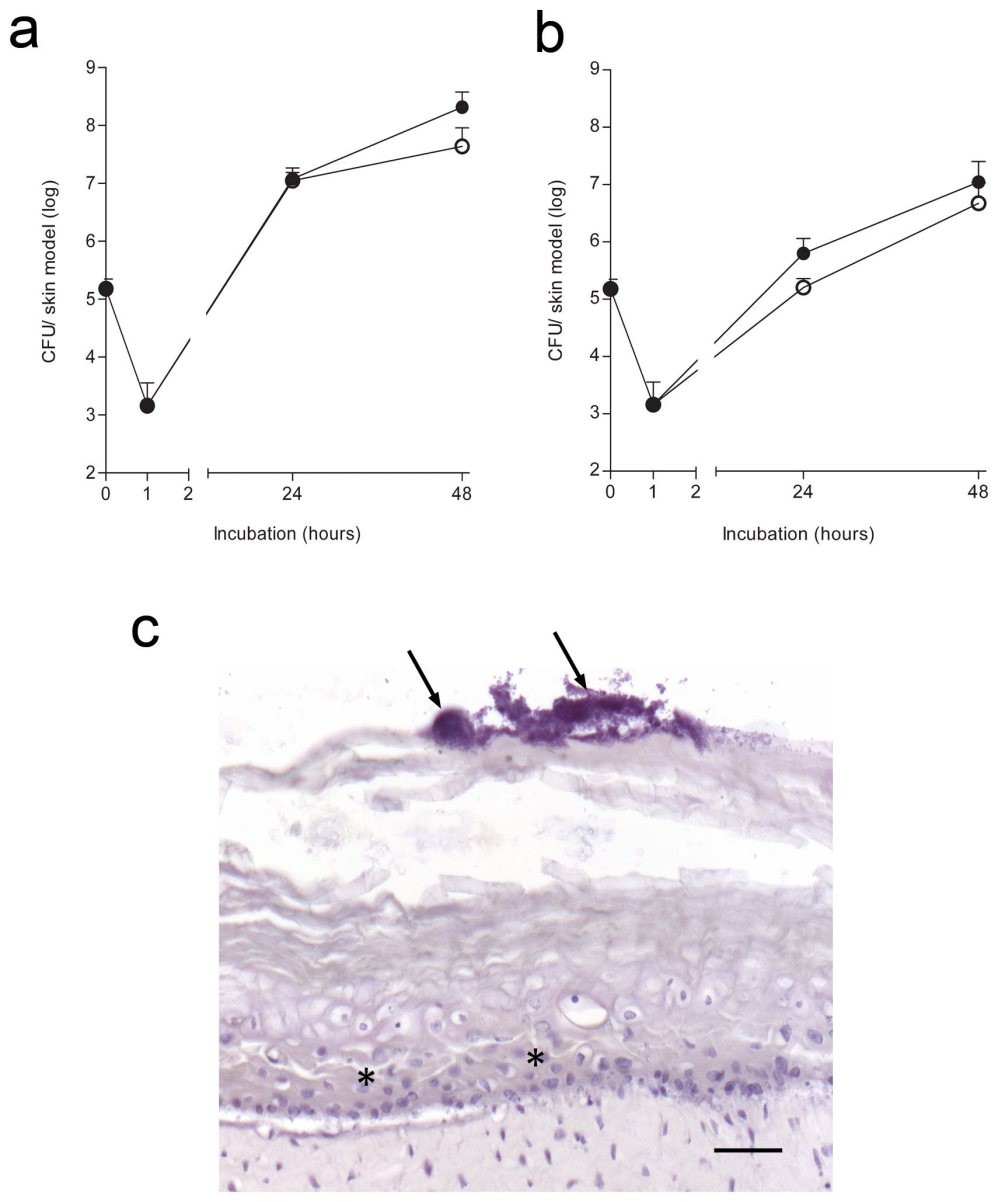
Course of MRSA growth on control and thermal wounded HSEs. The number of (**a**) detachable MRSA and (**b**) of tightly adherent MRSA to HSEs were assessed by the colony forming unit (CFU) assay (mean CFU/ml). Control (open circles) and thermal wounded (closed circles) HSEs were exposed for one hour to 1x10^5^ CFU MRSA, next the non-adherent bacteria were removed and at 24 and 48 hours thereafter. Results show the mean ± SEM. (**c**) Hematoxylin staining of thermal wounded HSEs exposed to MRSA for 48 hours. Arrows indicate bacterial biofilm and (*) indicates re-epithelialization. N=3. Scale bar = 50 μm.

Next, we compared the course of bacterial adherence and multiplication on thermally wounded and control HSEs by MRSA. After 1h of exposure to 1x10^5^ MRSA/HSE, less than 1% of topically applied bacteria adhered tightly to the epidermal layer of the HSEs (Figure 2a-b). Surprisingly, no significant difference was found in the increase in number of MRSA that were present on thermal wounded HSEs and control HSEs during the 24-48hrs after exposure (Figure 2a-b). In both thermal wounded and control HSEs, the number of MRSA that could easily be detached from the HSE was significantly higher than that of bacteria that became firmly adherent. On control HSEs, 1.5x10^5^ bacteria were tightly adherent compared to 3.5x10^7^ non-adherent MRSA (p=0.029) and for wounded HSEs 6.1x10^5^ MRSA were tightly adherent compared to 9.5x10^6^ non-adherent bacteria (p=0.022), (Figure 2a-b). Moreover, in both thermal wounded and control HSEs, small colonies were visualized on the stratum corneum, with haematoxylin staining (arrows in Figure 2c). Taken together, these results indicate that the course of adherence and multiplication of MRSA on thermal wounded and control HSEs after exposure to a fixed number of bacteria is quite similar. 

### Effect of thermal wounding and/or MRSA on Toll-like receptor expression by HSEs

**Figure 3:**
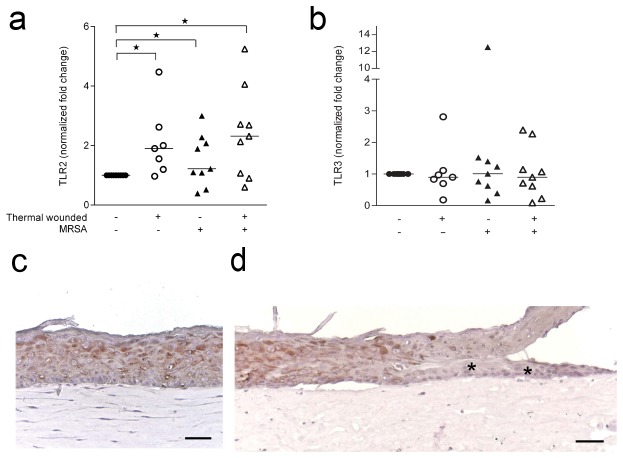
TLR2 and TLR3 expression by thermal wounded and/or MRSA exposed HSEs. The expression of TLR2 was assessed by quantitative PCR. (**a**) TLR2 mRNA expression in normalized fold change and (**b**) TLR3 by thermal wounded and/or MRSA exposed HSEs after 24 hours as compared to control HSEs. Horizontal lines represent the median. *P<0.05, as compared to control HSEs. (**c**) TLR2 protein expression in cross-sections of control HSEs. (**d**) Thermal wounded HSEs. N=6-8. * indicates re-epithelialization. Scale bars = 50 μm.

Because Toll-like receptors (TLRs) are involved in the cellular responses to wounding [18] as well as to bacterial presence [19,20], we assessed TLR expression in (MRSA exposed) thermal wounded and control HSEs. Expression of TLR2 mRNA was significantly enhanced in (MRSA exposed) thermal wounded HSEs, but not in control HSEs exposed to MRSA, as compared to control HSEs (Figure 3a). However, mRNA expression of TLR3 (Figure 3b) in HSEs did not significantly change after thermal wounding and/or the exposure to MRSA as compared to the expression in control HSEs. Specific immunostaining for TLR2 revealed that keratinocytes express TLR2 in the suprabasal layers of the epidermis (Figure 3c), while in thermal wounded HSEs the keratinocytes migrating over the wound bed lack TLR2 (Figure 3d, arrows). 

### Expression of inflammatory cytokines in response to thermal wounding and/or MRSA by HSE

**Figure 4:**
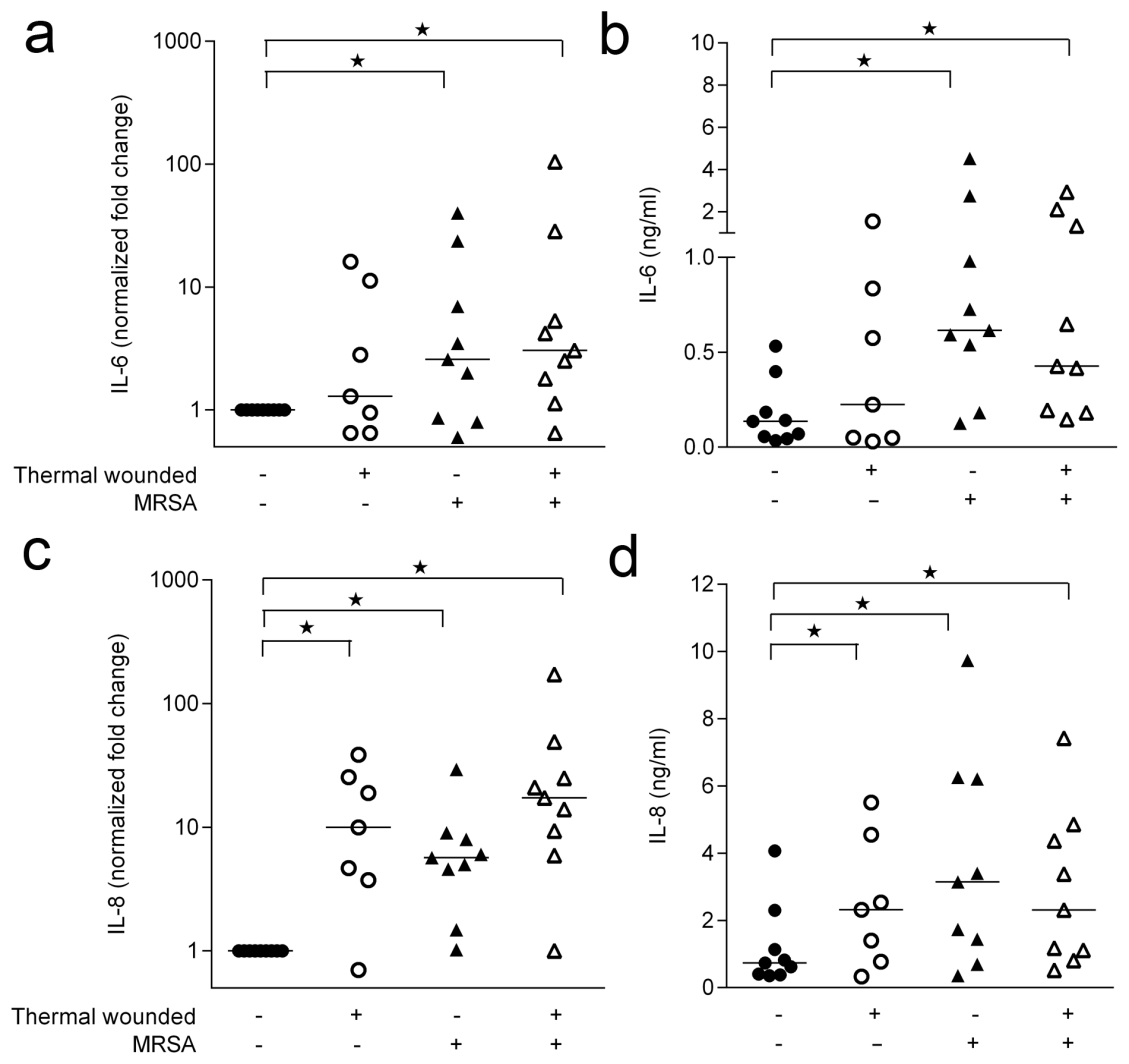
Presence of MRSA on HSEs leads to enhanced pro-inflammatory cytokine induction irrespective to thermal wounding. IL-6 and IL-8 expression by HSEs 24 hours after thermal wounding and/or MRSA was measured by quantitative PCR and ELISA. The (**a**) IL-6 mRNA expression (**b**) IL-6 protein production (**c**) IL-8 mRNA expression and (**d**) IL-8 protein levels of thermal wounded and control HSEs and HSEs thermal wounded and exposed to MRSA. N=7-9. * P<0.05, as compared to control HSEs.

As several inflammatory cytokines have been associated with skin wounding and the presence of bacteria/ bacterial components [16,21], we investigated whether IL-1α, IL-1β, IL-6, IL-8 and/or IL-10 were differentially expressed after thermal wounding and/or the presence of MRSA. Measurement of the cytokine levels in the HSE culture medium showed that thermal wounding did not induce expression of IL-6 (Figure 4a-b), while enhancing that of IL-8 (Figure 4c-d). Interestingly, the presence of MRSA resulted in significantly enhanced expression of both IL-6 and IL-8 by thermal wounded and control HSEs (Figure 4a-d). No IL-1α, IL-1β, and IL-10 was detected in the culture media of the various HSEs, irrespective of wounding or the presence of MRSA. However, mRNA analysis of thermal wounded as well as MRSA colonized HSEs showed increased IL-1β levels, but not IL-1α levels, as compared to control HSEs. Induction of Il-1β mRNA in thermal wounded HSEs and control HSEs by MRSA did not differ (Figure S1). 

### Expression of antimicrobial proteins by HSEs in response to thermal wounding and/or MRSA

Antimicrobial proteins, such as human β-defensin (hBD)-2, hBD-3, RNAse7 and LL-37 are associated with local antimicrobial activity, immunomodulation and wound healing [22,23]. Moreover, hBD-3 is specifically associated with *S. aureus* infection control [24]. We assessed the effect of thermal wounding and/or the presence of MRSA on the expression of these proteins in the HSEs. 

**Figure 5:**
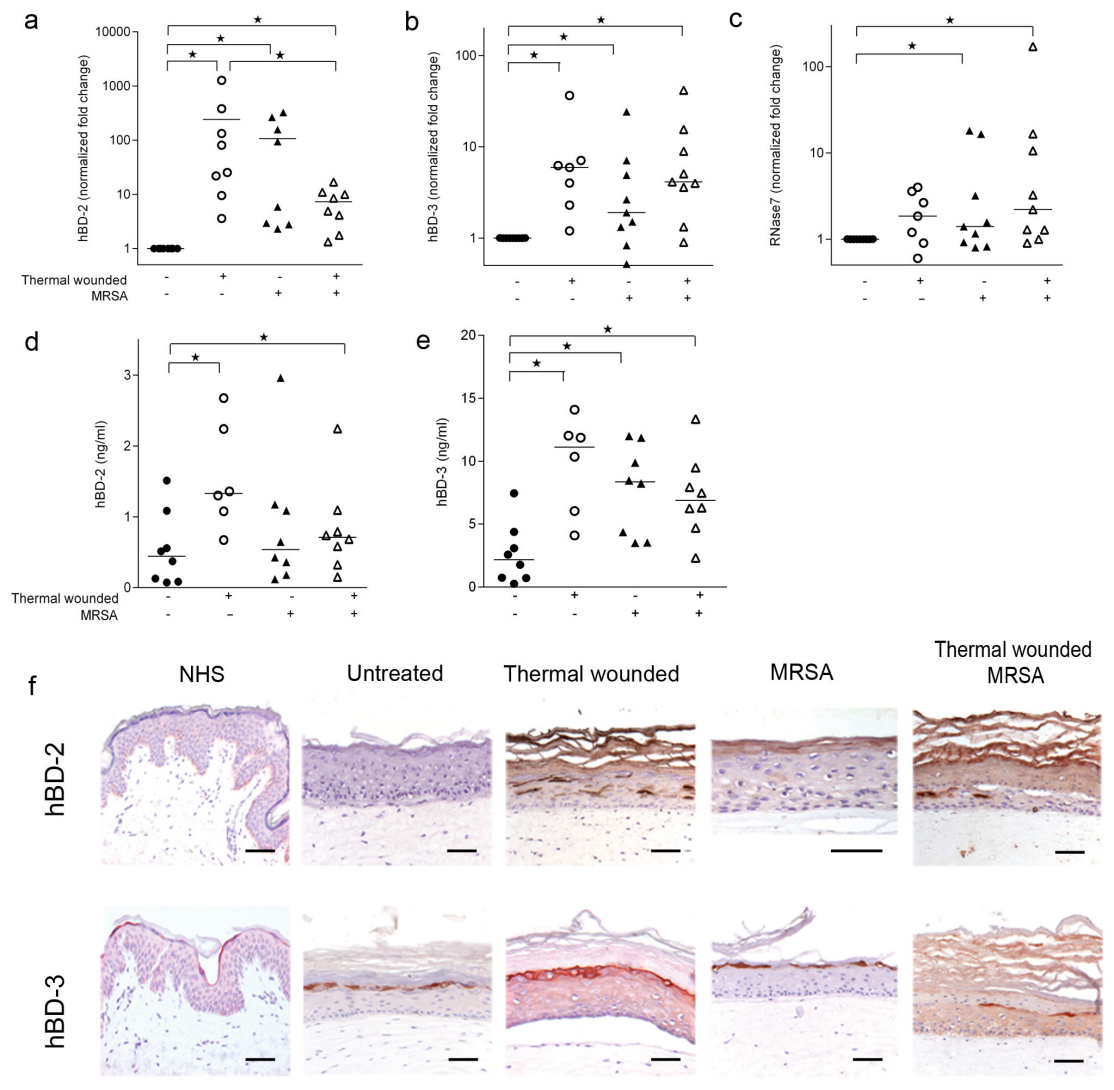
Antimicrobial protein expression by HSEs after thermal wounding and MRSA exposure. Expression of (**a**) hBD-2 mRNA, (**b**) hBD-3 mRNA, (**c**) RNase-7 mRNA, (**d**) hBD-2 protein and (**e**) hBD-3 protein by control, thermal wounded and/or MRSA colonized HSEs. *P <0.05. Bars are represented as the median. (**f**) hBD-2 and hBD-3 expression in normal human skin, control HSEs, thermal wounded HSEs, MRSA colonized HSEs, and thermal wounded and MRSA colonized HSEs. N=7-9. Scale bars = 50 µm.

Thermal wounding significantly enhanced the mRNA and protein levels of hBD-2, hBD-3 and RNase-7 (Figure 5a-e). Similar results were found the presence of MRSA on HSEs. Of note, the presence of MRSA on thermal wounded HSEs was not associated with enhanced levels of the various antimicrobial proteins as compared to thermal wounded HSEs without bacteria present (Figure 5a-e). Consistent with the mRNA data, tissue staining revealed hBD-2-producing keratinocytes in the stratum spinosum and stratum granulosum of thermal wounded and MRSA-colonized HSEs, but not in control HSEs and normal human skin (Figure 5f). In contrast to hBD-2, hBD-3-expressing cells were found in the stratum granulosum of thermal wounded HSEs, MRSA-colonized and control HSEs, as well as normal human skin (Figure 5f). 

The only member of the human cathelicidin family, LL-37, is generated by cleavage of the C-terminal end of the hCAP18 protein. We could not detect any expression of LL-37, in (MRSA exposed) HSEs. It was reported that LL-37 functions both as antimicrobial agent [25] and in immune modulation [26]. Supplementation of our culture system with 1,25-dihydroxyvitamin D3 induced both LL-37 mRNA and protein (Figure S2), confirming that HSEs can produce LL-37 provided that they are stimulated properly, e.g. 1,25-dihidroxyvitamin D3 [27]. 

### Effect of mupirocin on MRSA infected thermal wounded HSEs

**Figure 6:**
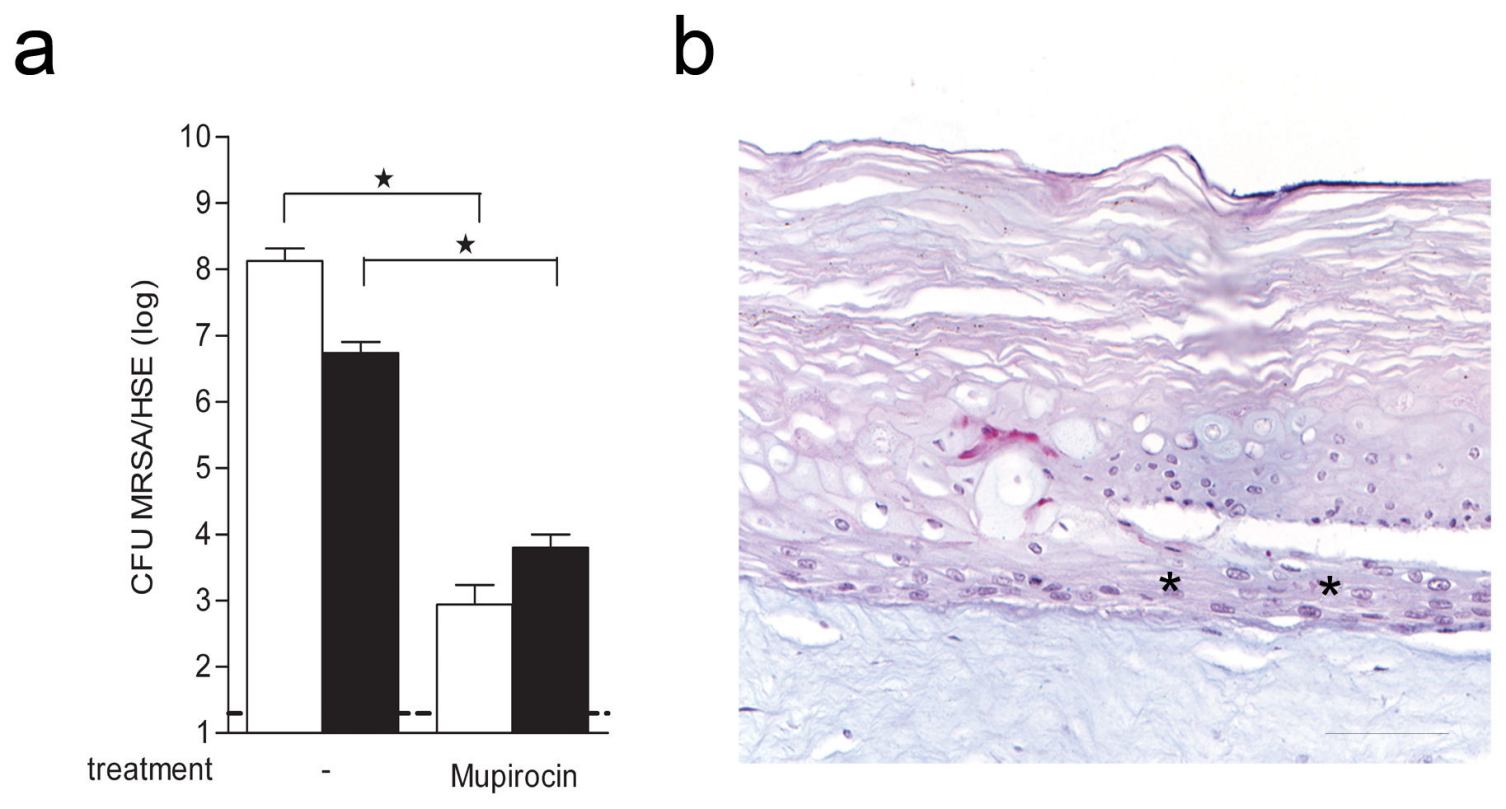
Eradication of MRSA from thermal wounded HSEs by mupirocin. (**a**) The number of detachable (white bars) and adherent bacteria (black bars) before and after mupirocin treatment were determined using the CFU assay. Results are expressed as mean CFU per HSE ± SEM. The dotted line represents the lower limit of detection. *P<0.05, as compared to untreated HSEs. (**b**) PAS/Alcian blue stained cross-section of mupirocin-exposed thermal wounded HSE that was infected by MRSA. * indicates re-epithelialization. N=3. Scale bar = 50 μm.

To explore whether antibiotics can be effective against the colonization of MRSA in the current wound infection model, we examined the effect of mupirocin on the numbers of MRSA on wounded HSEs. Results revealed that topical application of mupirocin (100 µg/HSE) significantly reduced viable counts for both detachable and adherent bacteria on HSEs (Figure 6a). In addition, mupirocin completely removed the bacterial biofilm from the thermal wounded HSEs without affecting the structure of the epidermis (Figure 6b). Together, these data indicate that the current *in vitro* wound infection model allows for the assessment of the local antibacterial effects of antibiotics. 

## Discussion

The present thermal wound infection model involving reproducible thermal wounding of HSE and subsequent exposure to MRSA, allows the assessment of the initial phases of the wound infection process in a human system. Furthermore, the effect of locally applied antimicrobial agents, such as mupirocin, can be assessed in this model. This conclusion is based on our following findings. First, thermal wounding of HSEs by nitrogen freezing is associated with keratinocyte and fibroblast death, detachment of the epidermis from the dermis, re-epithelialization of the epidermis and no enhanced keratinocyte proliferation. Secondly, adherence and subsequent multiplication of MRSA on HSEs, resulted in a less organized re-epithelialization as compared to intact models. MRSA did not penetrate the epidermis, indicating that the barrier function of the stratum corneum in this wound model is still effective. In agreement with this, the course of MRSA adherence and multiplication on thermal wounded and intact HSE was similar. Also, inoculation of (thermal wounded) HSEs with MRSA resulted in an inflammatory and protective immune response. Because we observe that the presence of multiplying MRSA triggered an immunological response in HSEs we decided to refer to our model as an in vitro thermal wound infection model. Finally, mupirocin effectively eliminated adherent MRSA, irrespective of biofilm formation, from thermal wounded HSEs, without damaging the model. 

The main advantage of this *in vitro* wound infection model is the usage of human cells to create skin equivalents that mimic the human skin, e.g. chemical properties stratum corneum [28] and cell differentiation [12]. This is in contrast to studies in animals where the skin structure may be very different from that of the human skin [9]. Moreover, the response of the host skin cells to the infectious agent and the response in the bacterium to its interaction with the human host can be studied simultaneously. In addition, skin equivalents survive longer than *ex vivo* skin biopsies and the experiments can be repeated with cells from the same donor. In contrast to others [29], we removed the fluid containing to non-adherent bacteria from the models to better mimic the dry environment of the skin [14]. The severity of the thermal wound infection in our models is considerably milder than that seen by Shepherd et al. who removed the complete epidermis leaving the dermis without a barrier [30], or than that of Werthen et al. who created bacterial biofilms in collagen matrixes [31]. 

The severity of thermal wounding in our HSEs could be increased by removing part of the epidermis or using mixed bacterial populations [32]. We observed a complete invasion of HSEs after 24 hours by MRSA with bacteria mainly residing between the dermal and epidermal compartment (data not shown). The introduction of immune cells, e.g. T-cells, Langerhans cells [33] and dendritic cells, in HSEs could provide additional information on the immune responses to MRSA in this model. However, the absence of an immune compartment in our HSEs enables the investigation into the direct effects of MRSA presence on a fully differentiated epidermis. 

An important finding of this study pertains to the similarities and differences in inflammatory and protective responses to MRSA between intact and thermal wounded HSEs. MRSA induced the expression of various pro-inflammatory cytokines, (IL-1β, IL-6 and IL-8) and antimicrobial peptides, (hBD-2, hBD-3 and RNAse7) in HSEs. This is in agreement with the findings by others who investigated keratinocyte monolayers exposed to inflammatory stimuli, heat-killed *S. aureus* and skin commensals [20,21,34] and in HSE after exposure to viable *S. aureus* [16,35]. Thermal wounding enhanced the expression of IL-8, hBD-2 and hBD-3. Moreover, after acute burn injury, human keratinocytes display an elevated pro-inflammatory cytokine expression profile [36]. It is reported that hBD-2 and -3 expression can be induced by superficial barrier disruption [22] are upregulated in the wound of burn wound patients [37]. However, in thermal wounded HSEs no further increase in the expression of these pro-inflammatory cytokines and antimicrobial peptides in response to MRSA colonization was observed.

We did not find any IL-1β protein in the supernatant of the HSEs. This could be due to the absence of the conversion of pro-IL1β to biological active IL-1β. Keratinocytes excrete pro-IL-1β upon stimulation, but to be biological active it has to be further possessed to IL-1β by proteolytic processing by caspase-1 [38]. After *S. aureus* infection pro-IL-1β has been shown to be processed by the Nlrp3 inflammasome in macrophages [39]. Since we did not find IL-1β in the culture supernatants of our HSEs, it could not be excluded that the Nlrp3 inflammasome is not formed by fibroblasts or keratinocytes present in the HSEs. 

The lack of responsiveness to MRSA of thermal wounded HSE could be due to components that are derived from wounded cells interacting with TLR3, this resulting in poor availability of this receptor to propagate inflammatory and protective responses [40–42]. Of note, we did not observe an altered expression of TLR3 after wounding and/or colonization by MRSA. TLR2 activation may mediate the induction of the inflammatory and antimicrobial mediators after thermal wounding [18] and MRSA exposure [43,44]. After thermal wounding, HSEs had an increased expression of TLR2 mRNA, irrespective of the presence of MRSA. This increased expression was similar to that of IL-8, hBD-2 and hBD-3. In contrast, the expression of IL-6 and RNAse7 was dependent on the presence of MRSA, while previously it was reported that RNAse7 expression was not induced by a gram-positive bacterial infection (e.g. with *S. aureus*) [24]. 

To investigate whether the current in vitro wound infection model is suitable for screening of new antimicrobial agents, we assessed the effect of the antibiotic mupirocin on MRSA infected HSEs. Mupirocin is used to eradicate Staphylococci like MRSA from the nasal cavity of people colonized with these bacteria [45]. We demonstrated that MRSA could be eradicated from wounded HSEs, using mupirocin. This indicates that our human HSE wound infection model is suitable to study the local effects of new topically-applied therapeutic agents on wound infections and healing. 

## Materials and Methods

### Cell cultures

Cell cultures of normal human keratinocytes (NHK) and fibroblasts (NHF) were established from fresh human mamma or abdominal surplus skin as described earlier [46,47]. All primary human skin cells from healthy donors used by the Department of Dermatology are isolated from surplus tissue collected according to article 467 of the Dutch Law on Medical Treatment Agreement and the Code for proper Use of Human Tissue of the Dutch Federation of Biomedical Scientific Societies. According to article 467 surplus tissue can be used if no objection is made by the patient. This means that the patient who will undergo plastic surgery is well informed on the research. In case he/she refuses the patient has to sign the inform consent form, if they agree they do not sign. This approach differs from other countries. None of the authors were involved in the tissue sampling and only birth date, gender and skin type of the subjects was known. The Declaration of Helsinki principles were followed when working with human tissue.

To obtain the cells, fat tissue was removed and the skin was incubated with dispase II (2.4 U/ml; Roche, Woerden, The Netherlands) after which the dermis and epidermis were separated. NHK were isolated from the epidermis after treatment with 0.05% trypsin (BD Falcon; Breda, The Netherlands) and cultured in DMEM (Invitrogen, Breda, The Netherlands) diluted 3:1 in Ham’s F12 (Gibco, Bleiswijk, The Netherlands) supplemented with 5% FCS (Hyclone/Greiner, Nurtingen, Germany), penicillin/streptomycin (100 U/ml-100 µg/ml; Invitrogen), 1 μM hydrocortisone, 1 μM isoproterenol and 0.1 μM insulin (all Sigma-Aldrich, Zwijndrecht, The Netherlands), further named keratinocyte medium. NHF were isolated after incubation in a 3:1 mixture of collagenase (Gibco) and dispase (Roche) for two hours at 37°C and cultured in DMEM supplemented with 5% FCS and penicillin/streptomycin, hereafter called fibroblast medium. 

### Generation of human skin equivalents

HSEs based on rat-tail collagen were generated as described earlier [13]. All rat-tail collagen used in this study was obtained from rat-tails dissected from cadaveric materials that would have been disposed of otherwise. The rat-tails were obtained from rats of the Animal Facility (Proef Dier Centrum) of the Leiden University Medical Center, The Netherlands. Rats came from the breeding facility, or were control animals of other studies and died by CO2 asphyxiation. Because no living animals were used for the purpose of this study, no approval was needed from the Institutional Animal Care and Use Committee. This is in agreement with the European directive for the protection of animals used for scientific purposes (2010/63/EU). 

In short, 3 ml of rat-tail collagen (4 mg/ml) was mixed with 1.25x10^5^ fibroblasts, pipetted into a 6-well filter insert, (0.4-μm filter Corning, Amsterdam, The Netherlands) and polymerized. This was cultured submerged in fibroblast medium for 2 days, after which NHK were seeded at 5x10^5^cells/filter. The cells were cultured under submerged conditions using keratinocyte medium supplemented with 1% serum, 1 µM selenious acid, 10 μM L-carnitine, 1 mM L-serine and a lipid mixture containing 25 μM palmitic acid, 15 μM linoleic acid, 7 μM arachidonic acid and 2.4 x 10^-5^ M Bovine Serum Albumin. After 2 days the HSEs were lifted to the air-liquid interface and cultured for an additional 10 days in serum free keratinocyte medium, containing 30 μM linoleic acid and supplements as described as above. All medium supplements were purchased from Sigma-Aldrich. 

### Thermal skin wounding

HSEs were thermally wounded as described earlier [13] with minor modification. In brief, wounds were created using a 2 by 10 mm blunt metal bar, further referred to as device, which was placed in liquid nitrogen for 2 minutes. Thereafter, the device was applied onto the HSEs for 15 seconds without any pressure. 

### Bacteriology and infection of HSE

MRSA, a clinical isolate, strain LUH14616 [48], was kindly provided by Dr. S. Croes (Maastricht University Medical Centre, Maastricht, The Netherlands). Inoculi from frozen cultures were grown overnight at 37°C on sheep blood agar plates (BioMerieux, Marcy l'Etoile, France). To colonize HSEs, LUH14616 was cultured for 2.5 hours at 37°C in Tryptic Soy Broth (Oxoid Limited, Basingstoke, United Kingdom) at 200 revolutions per minute. This suspension was diluted to 1x10^6^ CFU/ml using an OD600nm measurement, 100 µl of the suspension was pipetted onto the HSEs and incubated for 1 hour. Subsequently, the MRSA suspension was aspirated to remove non-adherent bacteria. At 24 and 48 hours thereafter the number of viable detachable and adherent bacteria was determined. HSEs were washed with 1 ml of phosphate buffered saline (PBS) and serial dilutions were plated on Diagnostic Sensitivity Test plates (Oxoid Ltd). To assess the number of viable adherent bacteria two punch biopsies (4 mm^2^) were taken from each HSE, homogenized in 1 ml of PBS and serially diluted for colony counting. The number of adherent bacteria per HSE (113.04 mm^2^) was calculated by multiplying the number of adherent bacteria in two biopsies (25.12 mm^2^) by 4.5. 

### Exposure of MRSA colonized HSE to mupirocin

HSEs colonized by MRSA for 24 hours were exposed to 100 µl mupirocin (1000 μg/ml; AppliChem GmbH, Darmstadt, Germany) in PBS or it’s diluent without antibiotic for 24 hours and then the number of detachable and adherent bacteria was determined as described above.

### Immunohistochemistry

(Immuno) histochemical analysis was performed on paraffin-embedded HSE sections. Slides (5 μm) were cut, deparaffinized, rehydrated, and washed with phosphate-buffered saline. Hematoxylin & Eosin (H&E) and PAS/Alcian blue staining (Dako) were performed according to instructions of the supplier. For immunostaining heat-mediated antigen retrieval using 0.01M citrate buffer at pH 6 was performed, followed by a block step using PBS/1% bovine serum albumin/2% normal human serum. For the detection of collagen type IV, the sections were treated with a 0.025% protease (Sigma) solution prior to antibody staining. Primary antibodies were incubated overnight at 4 °C. Thereafter, the tissue sections were incubated and stained with the avidin-biotin-peroxidase complex system (StrepAB complex/HRP, Dako) as described by the supplier. All tissue sections were counterstained with hematoxylin and embedded with Kaisers glycerin (Merck). 

### Antibodies

Keratin (K)10 (DE-K10, Labvision/Neomarkers, Fremont, USA), K16 (LL-025, ABD serotec, Kidlington UK), K17 (CK-E3, Novus biologicals, Cambridge, UK), Ki67 (MIB1, Dako, Glostrup, Denmark), TLR2 (TL2.1, Santa-Cruz Tebu-bio, Heerhugowaard, The Netherlands), collagen type IV (PHM12, Merck-Chemicon, Darmstadt, Germany), hBD-2 (R&D systems, Minneapolis, USA), hBD-3 (Abcam Cambridge, UK) and LL-37 (Innovagen, Lund, Sweden). 

### Enzyme-linked immunosorbent assay

Culture medium of the HSEs was collected and used to measure protein levels of IL-4, IL-6, IL-8, IL-10 (BioScource life technologies), hBD-2 and hBD-3 (Hycult biotech, Uden, The Netherlands). Protocol was carried out according to the supplier’s instructions. 

### Quantitative PCR

Total cellular RNA was extracted from epidermises of HSEs using the RNeasy Mini Kit (Qiagen, Hilden, Germany). During RNA extraction samples were treated with 27.3 Kunitz Units DNase I (Qiagen). Next, cDNA was synthesized from 200 ng RNA unsing the iScript cDNA synthesis kit (Bio-Rad, Veenendaal, The Netherlands) all according to the manufacturer’s instructions. PCRs were performed using the SYBR Green Supermix (Bio-Rad) and run on the MyIQ single color real-time PCR machine (Bio-Rad). Expression analysis was performed using the MyIQ software (Bio-Rad) and was based on the delta delta Ct method using household genes β_2_ microglobulin and Glyceraldehyde 3-phosphate dehydrogenase (GAPDH). The primer sequences are listed in Table S1.

### Statistical analysis

Data were analyzed by the Wilcoxon rank sum test for paired samples and Mann-Whitney test for unpaired samples using Graph-pad Prims 6.0 software package. P values of ≤0.05 were considered significant.

## Supporting Information

Figure S1 **IL-1α and IL-1β expression by HSEs 24 hours after thermal wounding and/or MRSA colonization was measured by quantitative PCR.** The (**a**) IL-1α mRNA expression and (**b**) IL-1β mRNA expression in normalized fold change. *P<0.05. N=7-8 experiments.
(TIF)

Figure S2 **MRNA and protein expression of LL-37 in MRSA exposed HSEs after 1,25dihydroxyvitamin D3 treatment.** (**a**) LL-37 mRNA expression in normalized fold change, light bar is 4 hours after treatment, dark bars is 24 hours after treatment. (**b**) LL-37 protein staining in MRSA colonized HSE after 24 hours. Arrows indicate LL-37 staining. Scale bar =50 µm.
(TIF)

Table S1 **Primers used for RT-PCR.** Reference genes are underlined.
(DOC)
