# Supplementary figures and images for: Inflammatory and Antimicrobial Responses to Methicillin-Resistant Staphylococcus aureus in an In Vitro Wound Infection Model
Source: PLoS One. 2013 Dec 10;8(12):e82800. doi: 10.1371/journal.pone.0082800 (PMC3858326; doi:10.1371/journal.pone.0082800)

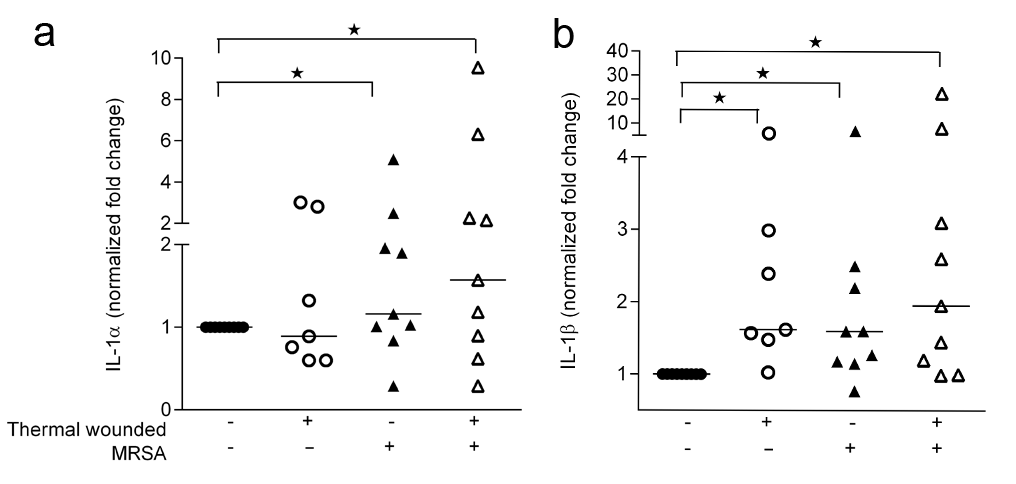

Supplement: Figure S1 — IL-1α and IL-1β expression by HSEs 24 hours after thermal wounding and/or MRSA colonization was measured by quantitative PCR. The (a) IL-1α mRNA expression and (b) IL-1β mRNA expression in normalized fold change. *P<0.05. N=7-8 experiments. (TIF) [file pone.0082800.s001.tif]

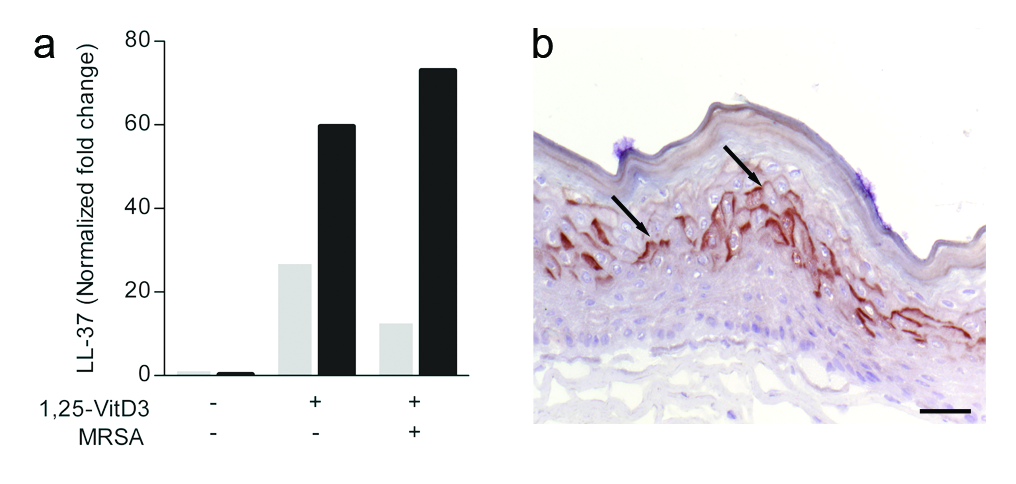

Supplement: Figure S2 — MRNA and protein expression of LL-37 in MRSA exposed HSEs after 1,25dihydroxyvitamin D3 treatment. (a) LL-37 mRNA expression in normalized fold change, light bar is 4 hours after treatment, dark bars is 24 hours after treatment. (b) LL-37 protein staining in MRSA colonized HSE after 24 hours. Arrows indicate LL-37 staining. Scale bar =50 µm. (TIF) [file pone.0082800.s002.tif]
