# Supplementary material for: Inflammatory and Antimicrobial Responses to Methicillin-Resistant Staphylococcus aureus in an In Vitro Wound Infection Model
Source: PLoS One. 2013 Dec 10;8(12):e82800. doi: 10.1371/journal.pone.0082800 (PMC3858326; doi:10.1371/journal.pone.0082800)
Supplement: Table S1 — Primers used for RT-PCR. Reference genes are underlined. (DOC) [file pone.0082800.s003.doc]

| **Gene** | **Sequence forward** | **Sequence reverse** |
| --- | --- | --- |
| **GAPDH** | AAGGTCGGAGTCAACGGATTT | ACCAGAGTTAAAAGCAGCCCTG |
| **β2M** | TGCTGTCTCCATGTTTGATGTATCT | TCTCTGCTCCCCACCTCTAAGT |
| **IL-1a** | CGCCAATGACTCAGAGGAAGA | AGGGCGTCATTCAGGATGAA |
| **IL-1β** | ACGAATCTCCGACCACCACT | CCATGGCCACAACAACTGAC |
| **IL-6** | GGTACATCCTCGACGGCATCT | GTGCCTCTTTGCTGCTTTCAC |
| **IL-8** | GCCAGGAAGAAACCACCGGAAGG | GGCTGCCAAGAGAGCCACGG |
| **TLR2** | GGCCAGCAAATTACCTGTGTG | AGCCGGACATCCTGAACCT |
| **TLR3** | TCCCAAGCCTTCAACGACTG | TGGTGAAGGAGAGCTATCCACA |
| **LL-37** | ATTTCTCAGAGCCCAGAAGC | CGGAATCTTGTACCCAGGAC |
| **hβD-2** | TGATGCCTCTTCCAGGTGTTT | GGATGACATATGGCTCCACTCTTA |
| **hβD-3** | TTATTGCAGAGTCAGAGGCGG | CGAGCACTTGCCGATCTGTT |
| **RNAse7** | GGAGTCACAGCACGAAGACCA | CATGGCTGAGTTGCATGCTTGA |
| **K16** | GAGATGCGTGACCAGTACGA | TTGTTCAGCTCCTCGGTCTT |
| **K17** | CATGCAGGCCTTGGAGATAGA | CACGCAGTAGCGGTTTCTCTGT |
